# Enhanced response rate to pegylated liposomal doxorubicin in high grade serous ovarian carcinomas harbouring *BRCA1* and *BRCA2* aberrations

**DOI:** 10.1186/s12885-017-3981-2

**Published:** 2018-01-03

**Authors:** Robert L. Hollis, Alison M. Meynert, Michael Churchman, Tzyvia Rye, Melanie Mackean, Fiona Nussey, Mark J. Arends, Andrew H. Sims, Colin A. Semple, C. Simon Herrington, Charlie Gourley

**Affiliations:** 1 Nicola Murray Centre for Ovarian Cancer Research, Edinburgh Cancer Research UK Centre, MRC IGMM, University of Edinburgh, Western General Hospital, Crewe Road, Edinburgh, EH4 2XU UK; 2 MRC Human Genetics Unit, MRC IGMM, University of Edinburgh, Edinburgh, UK; 3 Edinburgh Cancer Centre, Western General Hospital, Edinburgh, UK; 4 Division of Pathology, Centre for Comparative Pathology, Edinburgh Cancer Research Centre, MRC IGMM, University of Edinburgh, Edinburgh, UK; 5 Department of Pathology, Royal Infirmary of Edinburgh, Edinburgh, UK

**Keywords:** Ovarian cancer, BRCA1, BRCA2, PLDH

## Abstract

**Background:**

Approximately 10–15% of ovarian carcinomas (OC) are attributed to inherited susceptibility, the majority of which are due to mutations in *BRCA1* or *BRCA2* (*BRCA1/2*). These patients display superior clinical outcome, including enhanced sensitivity to platinum-based chemotherapy. Here, we seek to investigate whether *BRCA1/2* status influences the response rate to single-agent pegylated liposomal doxorubicin (PLD) in high grade serous (HGS) OC.

**Methods:**

One hundred and forty-eight patients treated with single-agent PLD were identified retrospectively from the Edinburgh Ovarian Cancer Database. DNA was extracted from formalin-fixed paraffin-embedded (FFPE) archival tumour material and sequenced using the Ion Ampliseq *BRCA1* and *BRCA2* panel. A minimum variant allele frequency threshold was applied to correct for sequencing artefacts associated with formalin fixation.

**Results:**

A superior response rate to PLD was observed in patients with HGS OC who harboured variants likely to affect BRCA1 or BRCA2 function compared to the *BRCA1/2* wild-type population (36%, 9 of 25 patients versus 12.1%, 7 of 58 patients; *p* = 0.016). An enhanced response rate was also seen in patients harbouring only the *BRCA1* SNP rs1799950, predicted to be detrimental to BRCA1 function (50%, 3 of 6 patients versus 12.1%, 7 of 58 patients; *p* = 0.044).

**Conclusions:**

These data demonstrate that HGS OC patients with *BRCA1/2* variants predicted damaging to protein function experience superior sensitivity to PLD, consistent with impaired DNA repair. Further characterisation of rs1799950 is now warranted in relation to chemosensitivity and susceptibility to developing ovarian carcinoma.

**Electronic supplementary material:**

The online version of this article (10.1186/s12885-017-3981-2) contains supplementary material, which is available to authorized users.

## Background

Ovarian cancer represents a substantial cause of mortality worldwide, with over 21,000 cases diagnosed, accounting for over 14,000 deaths, per year in the United States alone [1]. The majority of cases are ovarian carcinomas (OCs), approximately 10–15% of which arise in patients with inherited genetic susceptibility to disease [2, 3]. It is now recognised that the histologically-defined subgroups of OC represent distinct disease entities both molecularly and clinically, with high grade serous (HGS) OC accounting for the majority of cases (around 70%) [4].

Germline mutations in the DNA repair genes *BRCA1* and *BRCA2* (*BRCA1/2*) are responsible for the majority of hereditary OC, and around 15–20% harbour germline or somatic *BRCA1/2* defects [5, 6]. Mutational inactivation of *BRCA1/2* renders tumours deficient in homologous recombination DNA damage repair (HRR) [7, 8]. *BRCA1/2*-associated OC patients experience superior clinical outcome, despite their propensity for developing visceral metastases and a tendency to present with HGS histology [9–13]. These tumours display superior response rates to multiple lines of platinum-based chemotherapy, as well as superior sensitivity to PARP inhibitors, consistent with HRR-deficiency and dependence upon error-prone non-homologous end joining (NHEJ) to repair therapy-induced DNA damage [9, 14].

Pegylated liposomal doxorubicin (PLD) is a doxorubicin formulation, liposome-encapsulated and pegylated to increase drug half-life and reduce cardiotoxicity [15, 16]. PLD is often used in OC treatment in the advanced-stage, relapsed disease setting, with reported response rates of around 15% when used as a single agent [17, 18]. One mechanism of action of PLD is the induction of single-stranded and double-stranded DNA breaks through both free radical formation and direct intercalation into DNA, interfering with topoisomerase II-mediated repair [19].

A phase II trial comparing the PARP inhibitor olaparib at two doses versus PLD in a population of *BRCA1/2*-mutant patients with recurrent OC showed a greater than expected objective response rate to PLD [20]. Because *BRCA1/2* status is known to influence the response rate of patients to platinum-based chemotherapy, and induction of DNA damage is a common mechanism of action between PLD and platinum, we postulated that *BRCA1/2* status may also influence the response rates to PLD.

Three previous studies have attempted to address this hypothesis, but these investigations have suffered several methodological limitations [21–23]. All three studies include a number of untested “presumed *BRCA1/2* negative” OC patients in their wild-type comparator cohorts [21, 23]. Two studies included a significant number of patients treated with PLD in combination with other agents, most commonly platinum, which account for around half of the PLD-treated population in each study [21, 22]. One study limited *BRCA1/2* sequencing to regions of known founder mutations [22], and all three studies compared PLD response in a histologically heterogeneous population. Furthermore, these studies have limited sequencing to germline material, despite the substantial number of OC known to display somatic mutational inactivation of *BRCA1/2* [24, 25]. Given the known differential chemosensitivity of histological subtypes of OC [4], the clear potential for previous analyses to be confounded by superior response rate to co-administered platinum in *BRCA1/2*-associated OC [9], the limited predictive power of family history in predicting germline BRCA status in the presumed negative populations [26], and the known phenotypic overlap between germline and somatic *BRCA1/2*-inactivated OC [24, 27], there is a clear need for comparison of response rate to PLD monotherapy to tumour *BRCA1/2* status in a histologically uniform OC cohort.

Here we present next generation sequencing (NGS) of tumour DNA from a cohort of OC patients treated with PLD monotherapy from a single centre in order to better interrogate the interaction between *BRCA1/2* status and response to PLD.

## Methods

### Cohort identification and pathology review

**Fig. 1:**
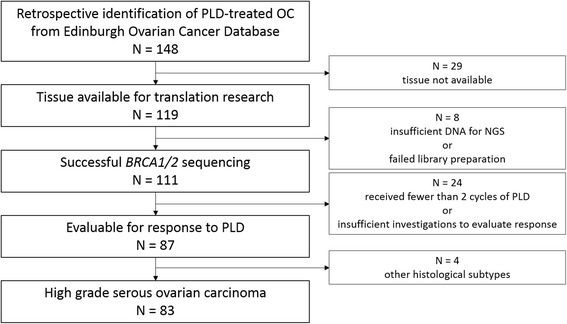
Flow diagram of HGS OC patients evaluable for PLD response

We retrospectively identified all patients treated with PLD monotherapy between 2001 and 2014 from the Edinburgh Ovarian Cancer Database (Fig. 1). 148 OC patients were identified. Of these, tumour material was available for translational research use in 119 cases. 10 μm sections were taken from archival tissue blocks alongside a 5 μm section to be stained with haemotoxylin and eosin (H&E). Ethical approval for the use of tumour material was obtained from South East Scotland Human Annotated Bioresource (East of Scotland Research Ethics Service Reference 10/S1402/33).

Tumour area identification and pathology review was conducted by an expert gynaecological pathologist using the H&E stained slide. Where histological subtype of OC was unclear from H&E alone, a combination of patient pathology reports and additional 5 μm sections immunohistochemically stained for WT1 and P53 proteins were used to determine OC histotype.

### DNA extraction

H&E stained slides were used to guide macrodissection of four 10 μm FFPE tissue sections per specimen. DNA extraction was performed using the QIAamp DNA FFPE Tissue Kit and Deparaffinization Solution according to the manufacturer’s instructions.

### NGS sequencing of *BRCA1 and BRCA2* in FFPE-derived tumour DNA

Sequencing of the *BRCA1* and *BRCA2* coding regions was performed using the Ion Ampliseq *BRCA1* and *BRCA2* panel on the Ion Torrent sequencing platform. 111 patients were successfully sequenced for *BRCA1* and *BRCA2*. BAM files were generated using Torrent Suite v4.6, and variants called using the Torrent Variant Caller v4.6.0.7. The minimum per-sample mean depth of coverage achieved was 916X; the median per-sample mean depth achieved was 4728X. The median uniformity of sequencing depth across targets was 90.5%. Called variants were functionally annotated using the Ensembl Variant Effect Predictor Version 75.

**Fig. 2:**
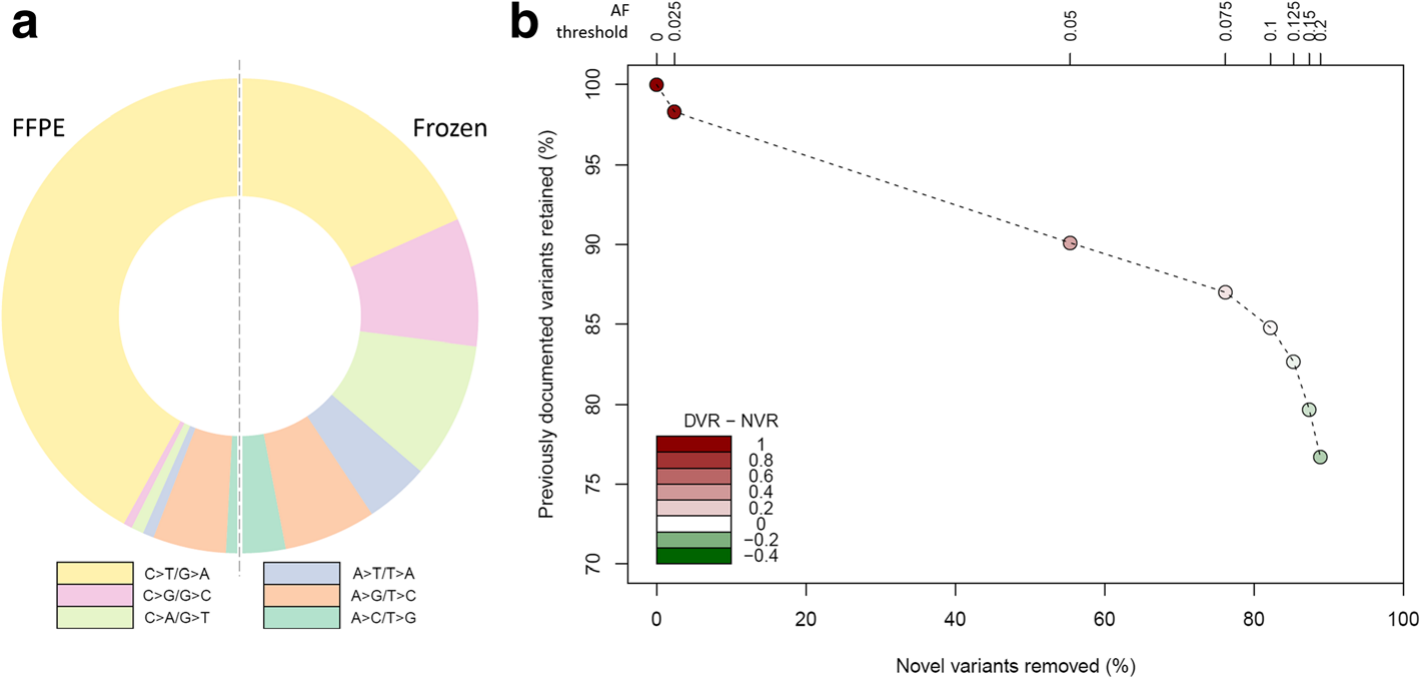
**a** Comparison of bi-allelic SNV spectra between DNA extracted from FFPE and fresh frozen material in the TCGA data. **b** Proportions of previously documented variants retained (DVR) and novel variants removed (NVR) at various minimum allele frequency (AF) thresholds

Sequencing of FFPE-derived DNA presents the challenges of both fragmentation and spontaneous deamination of DNA associated with formalin fixation [28, 29]. Consistent with these fixation artefacts, we observed a bias in the mutation spectrum of bi-allelic single nucleotide variants (SNVs) in our study compared to those reported in OC samples in the TCGA dataset, which utilised fresh frozen material (Fig. 2a) [25]. Consistent with previous reports, the strongest bias was in cytosine to thymine SNVs, likely as a result of cytosine deamination [29, 30].

To compensate for these artefacts, we applied minimum allele frequency (AF) thresholding to the set of variants. Comparing the proportion of previously documented (more likely true) variants retained to the proportion of novel (more likely false) variants, we found that for minimum AF > 10% more previously documented variants were lost than novel variants retained (Fig. 2b). We also compared the mutation spectrum of all retained bi-allelic SNVs at each AF threshold to the mutation spectrum from the fresh frozen TCGA samples, showing that the majority of the bias was removed at AF ≥ 10% (Table 1 and Additional file 1: Figure S1).

**Table 1:** Proportion of total SNVs accounted for by each SNV class at various minimum allele frequency (AF) thresholds and corresponding sum of squares differences (SSD) in SNV mutation spectra between FFPE and fresh frozen TCGA data

	Minimum AF threshold				Proportion of variants in fresh frozen data
SNV	no filter	0.05	0.1	0.15	
A > C/T > G	0.017	0.026	0.054	0.051	0.061
A > G/T > C	0.100	0.165	0.286	0.372	0.126
A > T/T > A	0.015	0.023	0.009	0.013	0.087
C > A/G > T	0.017	0.026	0.063	0.064	0.186
C > G/G > C	0.012	0.023	0.036	0.051	0.175
C > T/G > A	0.839	0.737	0.554	0.449	0.366
SSD	0.287	0.193	0.102	0.103	0.000

Together, these analyses demonstrated that a 10% AF threshold removed a large proportion of likely erroneous variants, while conserving the majority of likely true variants and minimising the difference in mutation spectrum compared to the TCGA data. Accordingly, variants detected at AF < 10% were discarded prior to analysis.

### Classification of functionally relevant *BRCA1* and *BRCA2* variants

Frameshift and nonsense variants in *BRCA1* and *BRCA2* were classified as likely damaging to protein function, as were previously reported missense mutations with known pathogenicity. Splice site variants with reported pathogenicity were also classified as likely to be damaging. Missense mutations predicted as unlikely to affect protein function by both Sorting Intolerant From Tolerant (SIFT) and PolyPhen scores were discarded as non-functional variants, while those predicted likely deleterious by both were classified as likely to be damaging [31, 32]. Missense mutations with conflicting SIFT and PolyPhen predictions were discarded as variants of unknown significance.

Three insertion/deletion (indel) variants called at high frequency across the cohort were identified as suspected recurrent sequencing errors around homopolymer regions. Sanger sequencing of these regions in the respective tumours confirmed these as sequencing errors, consistent with previous reports of false-positive indel calling around problematic genomic regions on Ion Torrent NGS platforms (Additional file 2: Table S1) [33].

### PLD response data

Patient response data were obtained retrospectively from the Edinburgh Ovarian Cancer Database. Responders were defined as patients who showed partial or complete CA125 tumour marker response or radiological response (either WHO or RECIST criteria as some patients predated RECIST reporting) from the PLD chemotherapy package. Patients who experienced stable disease, disease progression, or succumbed to disease on therapy were classified as non-responders. Patients for whom both CA125 data and scans were not available, or who received fewer than two cycles of PLD, were considered unevaluable for PLD response (Fig. 1).

## Results

### *BRCA1 and BRCA2* mutation frequency

Among the 111 successfully sequenced PLD-treated patients, 46 variants likely to affect protein function were detected, comprising 26 *BRCA1* variants and 20 *BRCA2* variants. Of the *BRCA1* variants, 12 were frameshift-inducing indels and 14 were missense variants, including 11 instances of the missense-causing SNP rs1799950 conferring a Gln356Arg amino-acid change and predicted to be detrimental to BRCA1 function by both SIFT and PolyPhen. Of the *BRCA2* variants, 10 were frameshift indels, 7 were missense variants, 1 was a nonsense mutation and 2 were splice site variants.

Across the study cohort 31.5% (35 of 111) of patients harboured at least one variant in *BRCA1* or *BRCA2* predicted as likely to affect protein function (*BRCA1/2*-aberrant). Specifically, 20.7% (23 of 111 patients) harboured at least one variant in *BRCA1* alone, 9.9% (11 of 111) displayed at least one variant in *BRCA2* alone, and 0.9% (1 of 111 patients) harboured variants in both *BRCA1* and *BRCA2*, consistent with previous reports of the higher *BRCA1* mutation frequency in OC versus *BRCA2* [10].

Of the *BRCA1/2*-aberrant population, 97.1% (34 of 35) were HGS OC, consistent with previous reports of the association between *BRCA1/2* mutation and HGS histology. The remaining case was high grade endometrioid OC.

### Patient demographics of *BRCA1/2*-aberrant and *BRCA1/2* wild-type populations

There was no difference in FIGO stage at diagnosis, success of primary surgical tumour debulking, platinum sensitivity at PLD therapy initiation, or in the number of lines of cytotoxic chemotherapy received prior to PLD between the *BRCA1/2*-aberrant and wild-type groups (Table 2). *BRCA1/2*-aberrant patients were significantly younger at diagnosis compared with wild-type (median 55 years vs. 64 years, respectively; Welch Two-Sample t-test *p* < 0.001), consistent with previous associations of *BRCA1/2* mutation with younger age at diagnosis [34].

**Table 2:** Demographic of PLD-treated patients

	*BRCA1/2*-Aberrant OC (*n* = 35)		Wild-Type OC (*n* = 76)		*p*-value
	No.	%	No.	%	
Age at diagnosis, years					
Median	55		64		<0.001^a^
Range	39–77		41–82		
Histology					
HGS	34	97.1	70	92.1	
Endometrioid	1	2.9	2	2.6	
Clear Cell	0	0	2	2.6	
Mucinous	0	0	0		0.429^b^
LGS	0	0	0		
Carcinosarcoma	0	0	2	2.6	
FIGO stage at diagnosis					
I	1	2.9	1	1.4	
II	3	8.6	4	5.6	
III	23	65.7	48	66.7	0.470^c^
IV	8	22.9	19	26.4	
NA	0	0	4		
Debulking status					
<2 cm	14	42.4	23	31.5	0.282^e^
≥2 cm	19	57.6	50	68.5	
NA	2		3		
Platinum sensitivity at PLD initiation					
Sensitive	5	15.2	9	12.5	
Resistant	28	84.8	63	87.5	0.761^d^
NA	2		4		
No. of chemotherapy lines prior to PLD					
≤2	25	71.4	61	80.3	0.429^e^
>2	10	28.6	15	19.7	
Evaluable for PLD response					
Evaluable	26	74.3	61	80.3	0.644^e^
Not evaluable	9	15.7	15	19.7	

^a^Welch Two Sample t-test; ^b^Fisher’s exact test, HGS versus non-HGS histology; ^c^Fisher’s exact test, early (I-II) versus advanced (III-IV) stage at diagnosis; ^d^Fisher’s exact test; ^e^Chi-squared test; NA, not available

### PLD therapy response rate

78.4% (87 of 111) of patients were evaluable for response to PLD. 19.5% (17 of 87) were classified as responders by virtue of achieving either a CA125 or radiological response. This observed response rate is comparable to that reported in other studies investigating the use of single agent PLD in the advanced-stage recurrent disease setting [17, 18, 35].

Different histological subtypes of OC are known to display distinct response profiles to chemotherapy [36–38]. The vast majority of patients classified as responders had disease of HGS histology (94.1%, 16 of 17), giving a response rate of 19.3% (16 of 83) in the HGS population. Given the limited number of non-HGS patients evaluable for PLD response in this cohort (*N* = 4), comparison of differential response rates to PLD between histological OC groups could not be made.

### *BRCA1/2* status influences response rate to PLD in HGS OC

**Fig. 3:**
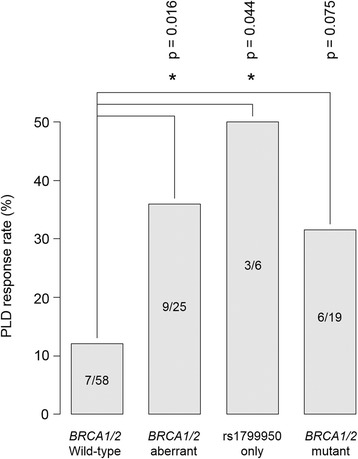
Differential response rate to PLD chemotherapy according to *BRCA1/2* status of sequenced tumour material. * indicates *p* < 0.05

We observed a significantly higher response rate to PLD in the *BRCA1/2*-aberrant HGS OC population compared with the wild-type group in this study (Fig. 3) (36.0%, 9 of 25 patients vs. 12.1%, 7 of 58 patients; Fisher’s exact test *p* = 0.016). Of the 9 responses in the *BRCA1/2*-aberrant population, two were radiological, 6 were CA125 tumour marker response, and one was both radiological and CA125 response. Of the 7 responses in the wild-type population, two were radiological, three were CA125 tumour marker response, and two were both radiological and CA125 tumour marker response. This *BRCA1/2*-aberrant population comprised both *BRCA1/2*-mutant patients and patients who harboured the predicted detrimental rs1799950 *BRCA1* SNP. When considering patients harbouring only rs1799950, we observed a significantly superior response rate to PLD versus the wild-type population (50%, 3 of 6 patients vs. 12.1%, 7 of 58; Fisher’s exact test *p* = 0.044), despite the small numbers of patients in this group (*N* = 6). There was a similar trend for superior response rate to PLD in the *BRCA1/2*-mutant population following the exclusion of patients whose tumour harboured only rs1799950 (31.6%, 6 of 19 patients vs. 12.1%, 7 of 58 patients; Fisher’s exact test *p* = 0.075).

## Discussion

Using NGS technology, we were able to sequence FFPE-derived DNA for the *BRCA1* and *BRCA2* genes in 111 PLD-treated patients at high sequencing depth. We observed a strong bias in the SNV spectrum of FFPE-derived DNA versus that reported by the TCGA study which utilised fresh frozen patient material, consistent with formalin fixation-induced artefacts known to occur in FFPE-derived DNA [29, 30]. We used a minimum allele frequency cut-off threshold for called variants to correct the mutation spectrum for these fixation artefacts. While this approach risks filtering a minority of true variants, we have demonstrated that it removes the bulk of fixation-associated artefacts, whilst retaining the vast majority of likely true positive variants, achieving a practical equipoise for variant filtering.

We detected 29 and 22 *BRCA1* and *BRCA2* sequence variants likely to affect protein function, respectively. Among these, we identified rs1799950, a *BRCA1* SNP predicted to be deleterious to protein function by PolyPhen and SIFT prediction tools. This SNP results in a non-conservative amino acid change in BRCA1 protein: the charged residue arginine is incorporated in place of the uncharged residue glutamine at amino acid 356. A previous study of high-risk prostate cancer families found that the minor allele of rs1799950 was associated with an increased risk of developing prostate cancer (OR 2.25, 95% confidence interval 1.21–4.20), but its relevance to treatment response is unstudied [39]. Furthermore, homozygosity of the minor allele state has previously been associated with breast cancer risk in a population from Saudi Arabia [40].

Of the 83 HGS OC patients evaluable for response to PLD chemotherapy, 19.3% responded to PLD, concurring with observations in previous studies [17, 18, 35]. Despite the low number of responders (*N* = 16), we were able to show a significantly superior response rate in those harbouring *BRCA1/2* aberrations predicted damaging to protein function, when compared to *BRCA1/*2 wild-type samples, of approximately 2.5-fold. This is consistent with the hypothesis that impaired HRR function renders OC sensitive to non-platinum DNA damaging agents such as PLD, as well as platinum-based chemotherapy.

A minority of patients harboured the *BRCA1* SNP rs1799950 with no other detected *BRCA1/2* defects. While this group were severely limited by size, we observed a greater than four-fold response rate in this population compared to the wild type population. Notably, this SNP was reported to possess a minor allele frequency of 0.0596 in European populations by the 1000 Genomes Project, and would therefore be considered a common variant according to population genetics conventions. These data suggest that rs1799950 is biologically significant in terms of response to cytotoxic chemotherapy, and further characterisation of this variant is now warranted. Future studies should seek to evaluate whether rs1799950, and other common *BRCA1* and *BRCA2* variants, modulate sensitivity to platinum and other DNA damaging agents in vitro, and address whether such variants convey inherited susceptibility to malignancy, particularly to OC and breast carcinoma.

Previous studies have shown that BRCA1-deficient OC are more likely to display high levels of tumour infiltrating lymphocytes (TILs) and display an enrichment of immune response genes [41]. OC with high levels of T cell infiltration have superior clinical outcome, thought to be secondary to an improved anti-tumoural immune response [42, 43]. Recent work has suggested that PLD may enhance the immune response in BRCA1-deficient tumours [44], and this may contribute to the improved benefit from PLD seen in *BRCA1/2*-aberrant tumours.

The higher response rate in *BRCA1/2*-aberrant patients presents an additional argument for prospective *BRCA1* and *BRCA2* sequencing in all OC patients. Given the low response rate to PLD in the *BRCA1/2* wild-type population (12.1% in our cohort), alternative therapies could be considered for the treatment of patients who have had germline or somatic *BRCA1* and *BRCA2* sequencing and have not displayed functionally relevant genetic changes in either of these genes. In light of the high response rate observed in *BRCA1/2*-aberrant patients, PLD should be considered as an active treatment option in patients with known *BRCA1/2* mutations.

Moving forward, the question remains as to whether this observed superior response rate extends to patients with defects in other components of the HRR pathway. In particular, detrimental variants in HRR genes known to be mutationally inactivated in a minority of hereditary OC – such as *BRIP1*, *PALB2* and *CHEK2* – may also predict response rate to PLD. Furthermore, the impact on PLD response rate, if any, of epigenetic silencing of *BRCA1* via promoter methylation remains unstudied.

## Conclusion

HGS OC patients displaying *BRCA1/2* sequence aberrations predicted detrimental to BRCA1 or BRCA2 protein function display an increased response rate to PLD. Patients harbouring the common *BRCA1* variant rs1799950 may also display a superior response rate to PLD. These data support the notion that HGS OC patients with *BRCA1/2* mutations are more sensitive to non-platinum DNA damaging agents compared to their *BRCA1/2* wild-type counterparts. The role of rs1799950 in chemotherapy sensitivity and predisposition to OC and BC warrants further investigation.

## Additional files

Additional file 1: Figure S1. Sum of squares differences (SSD) between our SNV spectrum and the fresh frozen TCGA SNV spectrum at various allele frequency threshold for variant filtering. (DOCX 53 kb)

Additional file 2: Table S1. Recurrently called variants confirmed as sequencing errors by Sanger sequencing. (DOCX 11 kb)

## Abbreviations

AF: allele frequency
FFPE: formalin-fixed paraffin-embedded
H&E: haemotoxylin and eosin
HGS: high grade serous
HRR: homologous recombination DNA repair
Indel: insertion/deletion
NGS: next generation sequencing
NHEJ: non-homologous end joining
OC: ovarian carcinoma
PLD: pegylated liposomal doxorubicin
SIFT: Sorting Intolerant From Tolerant
SNV: single nucleotide variant
