# Supplementary material for: Enhanced response rate to pegylated liposomal doxorubicin in high grade serous ovarian carcinomas harbouring BRCA1 and BRCA2 aberrations
Source: BMC Cancer. 2018 Jan 3;18:16. doi: 10.1186/s12885-017-3981-2 (PMC5753521; doi:10.1186/s12885-017-3981-2)

Supplementary Figure S1. Sum of squares differences (SSD) between our SNV spectrum and the fresh frozen TCGA SNV spectrum at various allele frequency threshold for variant filtering.


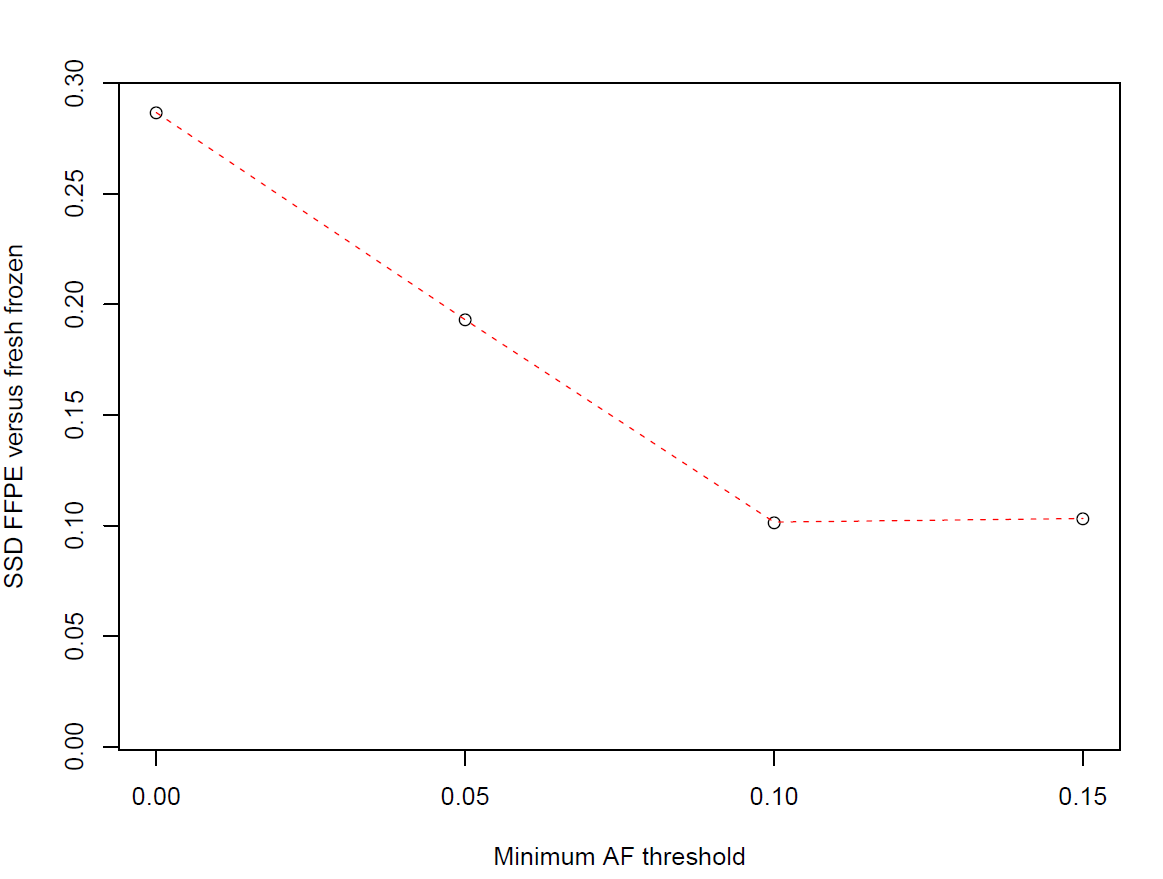

Supplement: Supplementary file 1 — Sum of squares differences (SSD) between our SNV spectrum and the fresh frozen TCGA SNV spectrum at various allele frequency threshold for variant filtering. (DOCX 53 kb) [file 12885_2017_3981_MOESM1_ESM.docx]
