# Supplementary material for: Enhanced response rate to pegylated liposomal doxorubicin in high grade serous ovarian carcinomas harbouring BRCA1 and BRCA2 aberrations
Source: BMC Cancer. 2018 Jan 3;18:16. doi: 10.1186/s12885-017-3981-2 (PMC5753521; doi:10.1186/s12885-017-3981-2)
Supplement: Supplementary file 2 — Recurrently called variants confirmed as sequencing errors by Sanger sequencing. (DOCX 11 kb) [file 12885_2017_3981_MOESM2_ESM.docx]

Supplementary Table S1. Recurrently called variants confirmed as sequencing errors by Sanger sequencing.

| **Genomic Location** | **Gene** | **Reference, Alt allele** | **Number of patients** |
| --- | --- | --- | --- |
| chr13:32907215 | *BRCA2* | GAA, GAAA | 11 |
| chr13:32906565 | *BRCA2* | CAAAAAA, CAAAAAAA | 4 |
| chr13: 32913676 | *BRCA2* | CAAAAAT, CAAAAAAT | 6 |
